# Impact of the COVID-19 Pandemic on Child and Adolescent Mental Health Policy and Practice Implementation

**DOI:** 10.3390/ijerph18189622

**Published:** 2021-09-13

**Authors:** Lawrence A. Palinkas, Jessenia De Leon, Erika Salinas, Sonali Chu, Katharine Hunter, Timothy M. Marshall, Eric Tadehara, Christopher M. Strnad, Jonathan Purtle, Sarah McCue Horwitz, Mary M. McKay, Kimberly E. Hoagwood

**Affiliations:** 1 Suzanne Dworak-Peck School of Social Work, University of Southern California, Los Angeles, CA 90089, USA; deleon@usc.edu (J.D.L.); easalina@usc.edu (E.S.); svchu@usc.edu (S.C.); 2 Office of Child and Family Services, Virginia Department of Behavioral Health and Developmental Services, Richmond, VA 23218, USA; katharine.hunter@dbhds.virginia.gov; 3 Office of Community Mental Health, Connecticut Department of Children and Families, Hartford, CT 06103, USA; tim.marshall@ct.gov; 4 Utah Department of Human Services, Substance Abuse and Mental Health, Salt Lake City, UT 84116, USA; erictadehara@utah.gov; 5 Office of Children’s Behavioral Health, Department of Children, Youth and Families, Providence, RI 02903, USA; christopher.strnad@dcyf.ri.gov; 6 Department of Health Management & Policy, Drexel University Dornsife School of Public Health, Philadelphia, PA 19104, USA; jpp46@drexel.edu; 7 Department of Child and Adolescent Psychiatry, New York University School of Medicine, New York, NY 10016, USA; Sarah.Horwitz@nyulangone.org (S.M.H.); kimberley.hoagwood@nyulangine.org (K.E.H.); 8 George Warren Brown School of Social Work, Washington University in St. Louis, St. Louis, MO 63130, USA; mary.mckay@wustl.edu

**Keywords:** COVID-19, mental health services, children and adolescents, telehealth, policy, implementation

## Abstract

Background: The impact of the 2019 coronavirus pandemic on the mental health of millions worldwide has been well documented, but its impact on prevention and treatment of mental and behavioral health conditions is less clear. The COVID-19 pandemic also created numerous challenges and opportunities to implement health care policies and programs under conditions that are fundamentally different from what has been considered to be usual care. Methods: We conducted a qualitative study to determine the impact of the COVID-19 pandemic on implementation of evidence-based policy and practice by State Mental Health Authorities (SMHA) for prevention and treatment of mental health problems in children and adolescents. Semi-structured interviews were conducted with 29 SMHA representatives of 21 randomly selected states stratified by coronavirus positivity rate and rate of unmet services need. Data analysis with SMHA stakeholders used procedures embedded in the Rapid Assessment Procedure—Informed Community Ethnography methodology. Results: The need for services increased during the pandemic due primarily to family stress and separation from peers. States reporting an increase in demand had high coronavirus positivity and high unmet services need. The greatest impacts were reduced out-of-home services and increased use of telehealth. Barriers to telehealth services included limited access to internet and technology, family preference for face-to-face services, lack of privacy, difficulty using with young children and youth in need of substance use treatment, finding a Health Insurance Portability and Accountability Act (HIPAA)-compliant platform, training providers and clients, and reimbursement challenges. Policy changes to enable reimbursement, internet access, training, and provider licensing resulted in substantially fewer appointment cancellations or no-shows, greater family engagement, reduction in travel time, increased access for people living in remote locations, and increased provider communication and collaboration. States with high rates of coronavirus positivity and high rates of unmet need were most likely to continue use of telehealth post-pandemic. Despite these challenges, states reported successful implementation of policies designed to facilitate virtual services delivery with likely long-term changes in practice. Conclusions: Policy implementation during the pandemic provided important lessons for planning and preparedness for future public health emergencies. Successful policy implementation requires ongoing collaboration among policy makers and with providers.

## 1. Introduction

The impact of the 2019 coronavirus (COVID-19) pandemic on the mental health of millions worldwide has been well documented [1,2,3,4]. A 2020 report from the Centers for Disease Control found that 46% of adults reported experiencing one or more mental or behavioral health problems [1]. The increased risk has been attributed to several factors, including economic losses and unemployment [3], fear of infection [2], and isolation and confinement [2]. Youth have been especially vulnerable to these impacts as family stress, prolonged isolation and confinement, separation from peers, home-schooling, fear of infection, grief over the loss of family members, and a profound sense of loss have contributed to elevated rates of depression, anxiety, and substance use [5,6,7].

Although the medical care system responded heroically and developed new procedures and medications to treat individuals with COVID-19, the impact of the pandemic on prevention and treatment of mental and behavioral health conditions is less clear. In particular, there have been no studies to date on the impact of the pandemic on policy implementation of child and adolescent mental health services. A few studies reported increased use of telehealth for mental health services delivery [8,9], but none have examined how successful these efforts have been or what policies have been developed to facilitate implementation. Such information is essential to learn how to prepare for and respond to future public health emergencies [10]. 

The COVID-19 pandemic has also created numerous challenges and opportunities to implement health care policies and programs under conditions that are fundamentally different from what has been considered to be usual care [11]. There have been several recent calls to apply principles and practices of implementation science to facilitate pandemic response and recovery [11,12,13,14]. Challenges to policy implementation during the pandemic include maintaining newly implemented practices that were instituted quickly without adequate infrastructures [11], forsaking fidelity for expediency [12] and utilizing standard practices for developing an evidence base under crisis conditions [14]. Although implementation science has been suggested as a means of improving COVID-19 responses related to testing and surveillance, contact and location tracing, public mask use and social distancing [13], it has yet to be applied to understand how state policy makers have and should respond to the policy demands resulting from the pandemic. 

In the context of an ongoing investigation of the implementation of evidence-based policy and practice for prevention and treatment of mental health problems in children and adolescents, we conducted a study to characterize public mental health agency policy makers’ perceptions of the impacts of the pandemic on youth and challenges to providing youth services during the pandemic, and to assess associations between these challenges and impacts [15]. A survey completed by 159 state/county mental health agency policy makers from 46 states in the September–October 2020 period found that policy makers perceived the COVID-19 pandemic as exacerbating youth mental health disparities, but not dramatically impacting the receipt of needed services. In this paper, we describe the findings of a qualitative study conducted simultaneously to determine the impact of the COVID-19 pandemic on implementation of evidence-based policy and practice for prevention and treatment of mental health problems in children and adolescents. Our aim was to obtain more detailed information on the current need and demand for services and capacity to deliver services and how State Mental Health Authorities (SMHAs) addressed COVID-related child mental health needs, with a particular focus on use of telehealth approaches to deliver mental health services.

## 2. Methods

Authors followed the Standards for Reporting Qualitative Research (SRQR) to report the findings from this study [16].

### 2.1. Participants

Eligible participants for individual interviews were representatives of State Mental Health Authorities (SMHAs) from 21 states in the United States. These representatives were selected from a random sample of 24 states stratified on the basis of the all-time coronavirus positivity rate at the start of this study (28 August 2020), taken from the Johns Hopkins Coronavirus Resource Center [17], and the mean rate of unmet need, defined as the percent of children, ages 3 through 17, with a mental/behavioral condition who did not receive treatment or counseling, taken from the 2017–2018 National Survey of Children’s Health [18]. These two criteria were selected because they were hypothesized to be associated with the supply and demand for mental health services. Potential participants were then contacted via email and provided with an information sheet describing study aims and procedures. Participants were provided with another key information sheet, both by email and verbally before beginning the interview. Twenty-nine state mental or behavioral health agencies responsible for delivery of mental health services to children and adolescents in 21 states (response rate = 87.5%) agreed to participate.

### 2.2. Data Collection

Using a semi-structured interview guide, all interviews were conducted online using the Zoom platform. Participants provided information on their agency’s mission and how that mission and associated activities had been affected by the COVID-19 pandemic. Specific questions related to the impact of the pandemic on services capacity, need and demand for mental health services among children and adolescents, plans developed to address the pandemic and evidence used to support planning, use of telehealth as part of these plans, barriers and facilitators to use of telehealth, and recommendations based on lessons learned. Interviews were conducted between late November 2020 and early May 2021, lasted between 45 min and one hour, and were recorded and transcribed for analysis. All study activities were reviewed and approved by the Institutional Review Boards of the University of Southern California and New York University.

### 2.3. Data Analysis

Consistently with recommendations for use of rapid assessment methods for qualitative data analysis [19], analysis of the data obtained from the semi-structured interviews followed a protocol embedded in the methodology of Rapid Assessment Procedures-Informed Community Ethnography (RAPICE) [20]. The method involves rapid data collection through semi-structured interviews and/or ethnographic fieldwork by clinically trained researchers, and data analysis by a trained qualitative methodologist, interviewers/fieldworkers and a group of SMHA representatives who were invited to serve as members of an Advisory Council to inform/clarify key themes and enrich descriptions. 

Analysis of interview transcripts and memos was conducted in two stages. In the first stage, transcripts were read, and key themes were elicited using the immersions/crystallization [21]. These preliminary findings were then presented to the SMHA Advisory Council comprised of State Mental Health Authority representatives, academic collaborators, and to the interviewers to gain more insight into the data and its context. In the second stage, focused thematic analysis techniques [22] were conducted beginning with assigning a priori and emergent codes to transcript text segments and using the method of constant comparison to construct key themes. Inter-rater reliability in the assignment of specific codes to specific transcript segments as part of the focused thematic analysis was assessed for five randomly selected transcripts. For all coded text statements, the coders agreed on the codes 98.6% of the time, indicating good reliability in qualitative research [23]. A discussion then ensued until both the research team and SMHA Advisory Council reached consensus as to the meaning and significance of the data. 

Questions in the semi-structured interview guide and thematic analysis of interview transcripts were based on constructs taken from two implementation frameworks, the Consolidated Framework for Implementation Research (CFIR) [24] and the Exploration, Preparation, Implementation and Sustainment (EPIS) framework [25]. Thus, the impact of the pandemic on services capacity focused on the inner setting of implementation; need and demand for mental health services among children and adolescents constituted the outer setting; plans developed to address the pandemic and evidence used to support planning highlighted the process of implementation; use of telehealth as part of these plans highlighted the characteristics of the intervention; and barriers and facilitators to use of telehealth illustrated both characteristics of individual participants (services providers and consumers, i.e., youth and families) and implementation process.

## 3. Results

Demographic characteristics of study participants are provided in Table 1 below. The average age of study participants was 49.2 years. Most of the participants were female (75.9%) and non-Hispanic White (82.8%) with a Master’s degree (62.1%) and serving as a director or deputy/assistant director of a state agency (24.1%) or division within the agency (33.5%). Coronavirus positivity rates at the time the interviews were conducted ranged from a low of 1.0% to a high of 26.1%, with a mean of 7.3%. Rates of children in need of mental health services who did not receive them ranged from 34.5% to 61.1%, with an average of 47.9%. The states were divided into four groups based on whether their positivity rate at the time of interview and percent of unmet need for mental health services fell above or below the respective medians for these two measures: high positivity/high unmet need, low positivity/high unmet need, high positivity/low unmet need, and low positivity/low unmet need. Mean rates of coronavirus positivity at time of interview and unmet need for mental health services by group are presented in Table 2.

Using the techniques of immersion and crystallization, analysis of the information contained in the interview transcripts revealed five broad themes that highlight both positive and negative consequences of the pandemic: (1) impact of the pandemic on need and demand for services; (2) impact on services capacity; (3) impact on child and adolescent mental health policy and planning; (4) process, challenges and consequences of telehealth implementation; and (5) recommendations for services delivery now and in the future. Each of these themes are described in detail below.

### 3.1. Impact on Need and Demand

SMHA representatives from 18 of 21 states (85.7%) participating in the interviews stated that the need for mental health services for youth experiencing depression, anxiety, and other mental and behavioral health problems had increased since the pandemic. Several participants acknowledged that the evidence was largely anecdotal; however, in several states, increased need was assessed indirectly via numbers of youth who have accessed mental health crisis hotlines, made emergency room visits, or were admitted to crisis stabilization units: *“And I think all of the crisis lines did experience an increase, and so I would say that does reflect an increase in need for mental health services”* (SMHA representative from a high positivity/high unmet need state). This need was attributed to increased levels of family stress, isolation and confinement, home schooling, fear of infection or family members becoming ill; and grief and sense of loss of family members and a way of life that existed before the pandemic.

*“I think, I think a lot of it is more stress, it’s not wanting to be quarantined and that the quarantine is really, really stressing our young people out. Sitting still, I think the families are carrying on the brunt of trying to be the teachers trying to work and their patience is low. There’s a lot of parent child conflict now.”*

In eight states, participants also reported that while the prevalence of youth with mild to moderate services did not appear to have increased, there was an increase in the acuity of mental and behavioral health problems that required inpatient hospitalization.

*“Mobile crisis teams visit people in their homes or community sites, and others meet clients in clinics or hospital emergency rooms. That is a dramatic drop for one quarter. Since then, the total usage of the services, the calls coming in has increased, but it is still a little bit lower than we normally experience. However, our acuity levels have been higher than they were in the past. Our ED visits have been high … While the total volume of kids seeking services has decreased, the acuity of those in need of services, those who require hospitalization or inpatient care, has increased.”*

Consistent with the perceived increase in need, representatives from 13 of the 21 states (61.9%) reported an increase in demand for mental health services. However, representatives from 12 of the 21 states (57.1%) reported a decrease in demand. These percentages do not add up to 100% because different representatives from the same state may have reported different patterns of change in demand or a single representative may have reported an increase in demand for one type of service and a decrease in demand for another type of service. For instance, in one high positivity/low unmet need state, a SMHA representative noted the following:

*“We have had differing effects in different programs that we run. For example, we have a program for trauma focused cognitive behavioral therapy and there has been a definite increase in the demand for the service, even though some of the kids really aren’t necessarily experiencing posttraumatic stress. In fact, you know, they’re, you know, the families are just, are justifiably, you know, stressed out and fearful of what is … of what, you know, what’s going to happen as a consequence of, you know, one of them getting sick or, you know, not being able to keep a second job.”*

However, none of the participants provided data on rates of depression, anxiety, substance use or any other mental and behavioral health problem that was independent of rates of services demand, making it difficult to determine percentages of youth in need who are not getting those needs met.

*“And then we also have data that our crisis line has had a dramatic increase in calls, but the data is a little … the data isn’t very clean, because the crisis line also accepts COVID related calls, so we haven’t yet distinguished between a personal emergency and a COVID related one. We don’t know … we’re speculating that there has been an increase in crisis calls for mental health, but, um, that’s a speculation. We don’t have the hard data yet until we can sort that out. But a lot of anecdotal stuff we’re hearing.”*

Moreover, while an increase in the number of calls to crisis hotlines had suggested an increase in need, there were also reports of a decrease in demand due to a lack of referrals from schools and reluctance of some families to use telehealth.

*“We have a program for more severe cases of psychopathology, namely, early psychosis, and because the schools were a key referral source for those cases, we are actually experiencing a tremendous drop. That’s, that’s an outpatient program, but we don’t have the kind of real reliable referral source of schools, especially for those schools that are not adopting a hybrid or some model where the kids are exposed to, you know, are accessible to school counselors or school social workers.”*

When examining variations by positivity and unmet need at the time of the interview, an increase in demand for services was reported by representatives from 8 of 10 states with high unmet need and 5 of 11 states with low unmet need, and by 8 of 10 states with high positivity and 5 of 11 states with low positivity (Table 3). The states with the highest percentage of participants reporting an increase in demand (100%) had high positivity and high unmet need. A decrease in demand for services was reported by representatives from 5 of 10 states with high unmet need and 6 of 11 states with low unmet need, and by 6 of 10 states with high positivity and 5 of 11 states with low positivity. The states with the highest percentage of participants reporting a decrease in demand (75%) had high positivity and low unmet need, while the states with the lowest percentage (25%) had low positivity and high unmet need. 

### 3.2. Impact on Services Capacity (Supply)

The pandemic has had both negative and positive impacts on the delivery of services. In 15 of 21 states, the greatest impacts have been on out-of-home services due to social distancing and reduced number of residential staff. These include inpatient hospitalizations, congregate care, and respite care. According to a participant in one high positivity/low unmet need state, *“because of safety procedures and social distancing, the number of available beds for these kids has decreased by 50 percent.”*

*“Um so yeah we had to close down our out of home treatment programs for a number of months. We froze admissions and the only we did make exceptions to that for us for emergency circumstances where youth had to transition. We just, we didn’t have a choice. We had to, from a harm reduction standpoint, we did admit a subgroup of kids, but our admissions really plummeted for about a six-month period. Um, let’s see, March, April, May, June, about five months and then we reopened admissions this past summer. And so, we saw a dip in the number of youth in our beds, some parents took their kids home because they didn’t want them in a congregate care facility, just for fear for safety reasons, and we saw a number of kids really struggling in the community who were referred for out of home but couldn’t get in.”*

Some inpatient settings and residential care facilities had to put a pause on intake admissions due to fewer numbers of available beds and because of staff sick leaves related to coronavirus infections, refusals to work out of fear of infecting family members, and staff burnout. In other facilities, wait-times for inpatient admissions were extended due to staff shortages.

*“And there have been periods of extended wait times for the psychiatric hospital based upon their workforce challenges of who may have been exposed or tested positive because, for example, they have three units, but they haven’t been able to open a third unit, which is a deficiency of about 18 beds because of not always having nursing or staff shortages have just hasn’t been consistent enough to fully be at capacity.”*

Some agencies lost revenue and staff due to fewer face-to-face services being provided. In nine states, the pandemic exacerbated a chronic shortage of trained providers and staff that has existed for years, as noted by the representative of one low positivity/high unmet need state: *“I think we have known that we needed to build up workforce for some time. And I think the impact of COVID has exacerbated that.”*

Many participants also reported diminished productivity of agency staff and services providers due to the increased stress they have experienced as a result of the pandemic. As one participant observed, *“Some people, you know, even on our staff being reduced to tears at this time because of the impact.”*

*“Our providers definitely have talked with us about feeling extremely maxed, exhausted at a level that they’ve never felt before. And it’s kind of a mix of staffing impacts because those staff need to care for their own children who are home learning or work and then be able to actually provide the direct service.”*

As with the difficulty identifying level of unmet need that can be attributed to the pandemic, some participants also reported difficulty determining whether unmet need was the result of reduced demand or reduced capacity. As noted by one representative from a low positivity/low unmet need state:

*“The other dynamic we’ve seen is our mental health providers have had a hard time filling positions. So, we’ve seen a lot of demand for mental health services and it’s unclear how much of it is going unmet because the demand has gone up or because they’re just not able to staff because pay rates are too low, the work is stressful. The demands during COVID have to have just thrown everything off um. So, there is absolutely unmet need um which I just I don’t know how much of it is due to because we’ve been having trouble filling positions versus a suspected spike in need, as well, I mean I think it’s probably both, but I don’t necessarily have the data to back that up.”*

Even in instances when services were being delivered to those seeking services, some SMHAs were not entirely convinced that they were receiving the services they needed; as expressed by one participant from a high positivity/high unmet need state, *“So there are children getting services, but that doesn’t necessarily mean that they’re getting the right services.”*

When examining variations by positivity and unmet need at the time of the interview, a decrease in capacity to deliver services was reported by representatives from 7 of 10 states with high unmet need and 6 of 11 states with low unmet need, and by 6 of 10 states with high positivity and 7 of 11 states with low positivity (Table 4). The percentage of participants reporting a decrease in capacity ranged from 50% to 75% in the four groups of states. Representatives of individual states indicated they had adequate staff but were encountering barriers to train them, lacked administrative resources to make the most of both the staff and available funding, or that those staff who continued to work were working harder.

Although most of the study participants identified features of the pandemic that negatively impacted service delivery, particularly out-of-home services and emergency services, many also identified positive outcomes associated with the pandemic. Representative of five states stated they experienced no significant impacts, and that every facility has been able to stay open. Benefits of the COVID-19 pandemic cited by participants included the destigmatizing of mental health problems and mental health services, an illustration of the need for an alternative system of service delivery, promotion of the use of schools to engage students and create more supportive environments, and a sense of pride in how the state mental health system adapted to the crisis. In one state, the pandemic created the opportunity to explore increasing capacity through provision of virtual services:

*“So that’s been a really positive thing, and you know I think has opened the door for conversations to allow more long term and ongoing options for treatment through virtual space that wasn’t allowed before. And using treatment services and so having those flexibilities have been good so we’re in a space now we’re trying to look at ongoing options for virtual utilization of virtual platforms or Tele health for youth in substance use treatment that previously wasn’t allowed.”*

### 3.3. Pandemic Policy and Planning

Representatives from six states indicated they had no specific plans related to pandemic response. However, state-level policy and planning efforts for these as well as the other 15 states revolved around two sets of priorities, delivering services during the pandemic and pursuing priorities identified before the pandemic. The first set of priorities reflected the impact of the pandemic on existing resources and services and efforts to deliver new services or expand on existing services. Representatives from six states identified the need to support both families and providers as a priority:

*“We have been trying to see what we can to support parents, and I gotta tell you, even our own staff who have young kids, it has been extremely stressful for them. So, in terms of a lot of our specific efforts have been aimed at our own operations. And we’ve been allowing an abundance of tele work as well as being extremely flexible. So, if a parent needs to sit with their kid in school in the morning we’re saying go ahead and do that just so we can maintain operations. So, a lot of it has been, how do we sustain our services during this time. I would say that has been a higher priority than what new services or interventions are we going to provide.”*

Representatives from five other states mentioned the need to maintain existing services like mobile crisis intervention, respite care, hospitalization, and substance use treatment. Delivery of new services included implementation of telehealth, helping schools deliver mental health services, and addressing stress and anxiety of parents and teachers, first responders, and frontline workers.

Suicide prevention was identified as one of the most important priorities that existed prior to the pandemic by representatives from six states.

*“Oh, suicide prevention was the other thing I was going to say with prevention. That is, you know, something certainly across the age continuum. [State]’s rate of suicide completion for 10 to 24 year-olds is above the national average. So that has been a focus, just like binge drinking, the same thing. So those two things were exacerbated. You know, during this COVID time. So, we’re working on kind of a statewide suicide prevention education awareness campaign, QPR, mental health first aid training. Some of those kinds of things. They always existed. But again, because of where COVID has impacted the pressure of young people and their families, that it is an obvious need and there were some young people that died by suicide that you know we just we just have to continue to do that … save lives. Okay?”*

Increasing equity is another priority that had existed prior to the pandemic.

*“Yes, so, um, a primary priority is to ensure continued increases to care access. And I should also note too, access to care in an equitable manner. To ensure that individuals across the state have that availability. We have a fairly large geographical landscape, but we also have a very small distribution of the population. So, the population that’s pretty heavily skewed in our urban area of our state. With a very, very small amount of our population in some of our bigger geographical areas which makes it challenging for them to get services over people in our more urban areas.”*

Other pre-pandemic priorities included workforce development, making certain services are evidence based, and building out continuum of care to insure comprehensive care.

Plans and policies to address the mental health needs of children and adolescents included identifying ways to provide more support for youth and their families, identifying ways to provide more support for schools and teachers, expansion of crisis counseling, and workforce development. In states representing high levels of coronavirus positivity and unmet need, plans to expand crisis services included developing a COVID crisis line for kids and staff and using paraprofessionals for crisis counseling. Plans to provide more support for schools included developing partnerships with schools to start psychotherapy groups or increase socioemotional learning environments. Other states were working on workforce development initiatives or expanding availability of behavioral health care in more settings. In states with low coronavirus positivity rates and high unmet need, planning and policies targeted developmentally appropriate youth, mobile crisis programs, respite care, family peer support, workforce development initiatives, inter-agency collaboration around suicide prevention, delivering care to high acuity clients across systems, increasing use of evidence-based practices for services delivery, increasing compensation for services delivered, increasing public awareness of mental health needs and available services, and increasing access to services. In states with high rates of coronavirus positivity and low rates of unmet need for mental health services, SMHAs were involved in planning and policy making to increase capacity to build out the continuum of care for youth and families, deliver substance use treatment services, and expand home visiting and service delivery in rural areas. In states with low levels of coronavirus positivity and unmet need, the three major programmatic and planning emphases were placed on identifying ways to provide more support for youth and their families, like creating a website with a curated set of resources for parents of elementary school age children; identifying ways to provide more support for schools and teachers; policies and plans that address capacity to deliver services; and providing guidance to providers for delivering services impacted by the pandemic, including crisis services, residential treatment, and home-based services. 

In developing these plans, participants most frequently engaged with representatives from other state agencies (*n* = 16), including public health, child welfare, and juvenile justice; providers (*n* = 14); educators (*n* = 12); consumer advocates (*n* = 11); health care system administrators (*n* = 6); parents/caregivers (*n* = 7); and youth (*n* = 4). Engagement with stakeholders appears to be greater in states with low coronavirus positivity and low unmet need (4.9 groups per state) than in states with high positivity and high unmet need rates (2.8 groups per state). 

The most frequent source of evidence used in developing these plans across all states was administrative data collected by the participant’s agency or other agencies within the state (*n* = 8), followed by feedback from providers (*n* = 5), and information on the experience of other states (*n* = 7). Other evidence included searches of the research literature (*n* = 5), access to national data sets (*n* = 3), and review of national standards (*n* = 3). This evidence was obtained from provider calls (*n* = 5), other state agencies (*n* = 5), federal agencies (*n* = 2), non-profits (*n* = 2), intermediary organizations (*n* = 3), university partners (*n* = 2), professional associations (*n* = 1), and internal experts (*n* = 2). The quality of evidence was evaluated on the basis of confidence in the data (*n* = 3), evaluation by external experts (*n* = 4), acquaintance with the source of the evidence (*n* = 3), consistency with experience (*n* = 1), and rapid turnover of request for evidence (*n* = 2). There did not appear to be any noteworthy variations in use of evidence by positivity rate or rate of unmet need. 

### 3.4. Implementation of Telehealth

Perhaps the greatest challenge to delivering mental health services during the pandemic was barriers to the use of telehealth services. Representatives from 10 states reported having a telehealth system structure for delivery of health services prior to the pandemic, but it was rarely used for delivering mental health services to children and adolescents, largely because such services were not reimbursable. Representatives from almost all (*n* = 20) states reported limited access to broadband and internet services, especially in rural areas, and limited access to technology needed for telehealth, including lack of laptop computers and limited minutes with cellphone plans. Fourteen participants reported that some families were reluctant to participate because they were unfamiliar with the practice, did not feel comfortable using the technology, were concerned about privacy, preferred face-to-face interactions with providers, and because over time they began to experience virtual fatigue. Youth with psychoses and/or living in unsafe environments with little privacy were also reluctant to use telehealth services. Participants from 11 states also mentioned reports from providers that telehealth services were difficult to use with very young children, clients with more severe problems, and with clients in need of substance use treatment.

*“I think the, the biggest challenge is families’ access to broadband and access to good devices, you know, you might have a family that they’ve got to one computer, or one tablet and it’s like there’s big problems with people having the privacy to do a session over the one piece of equipment when you know the brother needs that for his class or there’s no … there’s no place in the house to go to have a session where there aren’t people around.”*

*“This is sort of technological. Some of the parents are kind of techno phobic. And they don’t they don’t like to … they feel like ‘I don’t know how to do this. It’s too much trouble …’* *But we have surveyed our families and the majority of them are okay with telehealth, but the majority of them prefer in person, if that makes sense. So, I think, I think there’s less of an engagement when it comes to younger children. My people are telling me that it’s harder to engage young kids through tele health.”*

Other barriers included finding a platform that was HIPAA-compliant (i.e., that, in accordance of the Privacy Rule of the Health insurance Portability and Accountability Act, calls for the protection from unlawful disclosure of “individually identifiable health information”, including demographic data, that relates to the individual’s past, present or future physical or mental health or condition; the provision of health care to the individual, or the past, present, or future payment for the provision of health care to the individual, and that identifies the individual or for which there is a reasonable basis to believe it can be used to identify the individual [26]), provider access to technology, training providers how to deliver services and clients how to receive them, provider indecision as to which platform to use, a reduction in the number of hours meeting with clients because of the demands of receiving services virtually on clients, and challenges in getting private insurance companies to reimburse for telehealth services.

The distribution of reported barriers to use of telehealth by rates of coronavirus positivity and unmet need for child and adolescent mental health services is provided in Table 5. States with high positivity and low rates of unmet need for child and adolescent mental health services reported slightly more barriers (5.14 per state) than other state groups.

To address these barriers, the use of telehealth or other virtual platforms also required changes in state guidelines or policies regarding mental health service delivery. Representatives from nine states mentioned policy changes required to enable reimbursement for services. This included changes in Medicaid payment policies, private insurance payment policy, and some regulations based on federal guidelines. One participant stated that the Federal government suddenly stopped the COVID emergency measures and then reimbursement for telehealth stopped for a time. Representatives from more than one state each mentioned policy changes to allow the use of telehealth to deliver substance use treatment services, licensing of providers to deliver services via telehealth, including providers living in other states, and using platforms that ensures privacy and were HIPAA- compliant. Other changes that were cited by representatives from individual states included eliminating the requirement that a client’s first visit for assessment had to be face-to-face, changes in regulations regarding tele-supervision of providers, issuing standards of care for telehealth, and addressing internet connectivity for use of telehealth. Most of the policies were premised on immediacy, flexibility and the availability of funding.

*“So, traditionally, pre COVID you know, we couldn’t provide tele supervision necessarily or, you know, we couldn’t have too many hours of Tele supervision to meet licensure requirements, and then we also couldn’t bill for Tele therapy, the only exception to that was if you had telemed qualifications. So, all of those got waived in an emergency way, to the point that we could even use facetime.”*

*“Yeah, there have been changes in Medicaid payment policies, insurance payment policy, some regulations. Like initially, you know, it was starting to change before the pandemic, thank goodness. But even just a year, year and a half ago, there were rules, Medicaid rules where it’s like the person doing tele health had to be another licensed clinics space and all these really incredible regulations and that’s all gone. So just so it’s much more flexible.”*

Despite the barriers to implementation cited above, the transition to telehealth was considered by participants to have been perhaps the most positive outcome of the COVID-19 pandemic. As described by the SMHA representative of one low positivity, low unmet need state:

*“Early on, we were amazed at how upbeat our providers were and how quickly they pivoted from face-to-face to virtual encounters with their clients. The transition was completed in about 3 to 4 weeks. We also realized that we were meeting more frequently with our program managers. We normally would have monthly meetings but that shifted to weekly when we started meeting virtually. Most of our meetings transitioned back to face-to-face, but with the recent resurge in COVID cases, we have moved back to virtual meetings.”*

Seven participants reported that telehealth services resulted in substantially fewer appointment cancellations or no-shows and greater family engagement, especially because transportation to and from the provider’s office was unnecessary. In one state, no show rates were reported to have declined by 50%. Use of telehealth to deliver services also reduced the amount of time traveling to provide services and reduced provider risk of infection. Participants from 12 states also reported that adolescent clients in particular appeared to prefer telehealth to in-person services delivery and services appeared to be as effective if not more effective. Access to services has now increased due to telehealth for people living in remote locations. Going virtual has increased communication and collaboration with providers. Although session duration was shorter when compared to in-person visits, telehealth visits were more frequent. Social media promoting telehealth has resulted in an increase in demand. Participants from one state reported that providers had been creative in using telehealth with younger clients.

*“In some ways, with the increased access to Tele health or audio health I think we’re perhaps reaching populations that maybe we didn’t prior, when they had to walk into a building or the services … time in place, but I also think for certain populations, there’s probably less access and service. I know for our residential, both mental health and substance use, with COVID that’s a challenge.”*

Factors that facilitated the use of telehealth included provider training in its use, information for families on its use, grant funding to provide client access, purchase by SMHAs of cell phones and laptops computers for client and provider use, laptops provided by schools, use of prepositioned infrastructure, availability of platforms to clients and providers, online support groups, federal guidance on how to get around HIPAA restrictions, telephone service providers who made adapted plans available to families, an ability to secure billing for services, and the occurrence of a cultural shift to using telehealth for mental health services shared by providers and families.

*“I think that, you know, the schools all gave out laptops. So, you could pick up a laptop for free. And so, families that may not have had access previously had access. We have two agencies, and they may not be the only ones, but [a state parents association] posted quite a bit of information early on about helping kiddos and helping families … There’s several different entities that are getting information out there to families about the effectiveness or the ability to use tele health and access to care. And so, I’d say there are other entities, besides just the Department of Health and Welfare, that really stepped up to try to help kids and families to provide that information and encourage them to use telehealth as a way of getting mental health resources.”*

*“I would also add that, especially early on in the pandemic, we accessed some CURES grant dollars, we also reallocate at some of our state general funds to support providers in purchasing the resources to provide services remotely so that we still continue to get requests for equipment such as you know, the video cameras for computers or laptops or even like wi fi little jet packs for the families themselves, for their clients.”*

*“I think in general too there’s just been, you know, a societal and culture shift to people being more open and we’re having to be more open to using the Internet and the phone to access really anything these days. So, I think that like just even that culture shift has changed things a lot to people’s willingness to go that route.”*

However, the reliance on telehealth had mixed results with respect to issues of equity. On the one hand, telehealth was perceived to improve access to care, especially in rural states where the number of providers was limited. As one of the participants from a predominately rural state observed, “*we may be able to see more children in the frontier areas that are receiving services.”* Another state representative noted: “*Access, showing up, not canceling, all those barriers, you know childcare barriers, transportation barriers, distance barriers. They go away for many people …”* On the other hand, limited access to technology and the internet was perceived as exacerbating pre-existing disparities in access to care. As described the SMHA representative of one low positivity, low unmet need state:

*“As with everywhere, [our state] is a pretty diverse place socio economically, and I think, as happens everywhere, families who are better off are generally also do better with things like Tele health, but our families who are not … don’t have kind of seamless wi fi access or the devices or whatever it like I think the ability to connect is harder, you know when you have a family that’s using their pre purchase minutes to do a session like. And you know we’ve actually tried to push out to our providers funding so that they can support Tele health, so if they need to get whatever device for someone they can. We’ve made a decent amount of funding available for that, I think that is good, however there’s just like … it’s hard enough to engage some families and then, when you put in the connectivity, on top of it, I think it is exacerbating kind of the racial and ethnic disparities. COVID has not helped with that at all as well. I think it is also harder … you know we are … [State’s] mental health system is largely … the professionals in the system are largely white and a lot of our state is still majority white um, but I think we are about 40% people of color and there’s just … COVID is just exacerbating that dynamic as well in really problematic ways and so it’s much harder to engage families and especially over Tele health.”*

Because of the positive elements associated with use of telehealth for service delivery, representatives of 17 states (81.0%) indicated a preference of continued use of these services post-pandemic. A survey conducted in one state reported that 64% of service providers said they wanted to continue delivering services via telehealth. Other participants indicated they would continue it use if it was billable. Participants were encouraged to continued use because it seemed to be popular with youth and families, and because appointments were being kept. Representatives of a few states expressed reservations to continued use since their system of care had been dependent on face-to-face delivery of services.

*“And I think, I think the early research on tele health has shown that it’s just as effective as a person and I think there are exceptions, of course, so I’m hoping that it becomes a large part of our service repertoire, but I do hope that people don’t forget about the value of in person services and go back to doing a large amount of that as well, especially our in-home services. I think you’re in the family’s environment and that can be really helpful in intervening on problematic behavior to know what it’s like to be in that house. So, so I you know there’s another thing I can tell you is that there’s been a decrease in no show rates.”*

*“And I think providers will continue to use Telehealth. I think it’s something that they have found as effective. I think overall, I think people especially kids are pretty comfortable that talking on a, you know, that’s what they’re used to. And so, I think in the long run, there may be even more effective work that’s being done through telehealth, just because I think kids will feel more comfortable than walking into a doctor’s office and having to talk to a real person, not something they like to do very much.”*

*“I believe they will because they have seen the benefits and if it is allowed to be a billable service, they will continue it to the extent that it is allowable.”*

When states were compared by rates of coronavirus positivity and unmet need, the percentage of states reporting positive outcomes with telehealth implementation ranged from 80% to 100%, the latter in states with high positivity and high unmet need and states with low positivity and low unmet need. A desire to continue use of telehealth post-pandemic ranged from 60% to 100%, the latter in states with high rates of coronavirus positivity and high rates of unmet need.

### 3.5. Recommendations for Services Delivery

Every participant provided one or more recommendations for providing mental health services to children and adolescents they might provide to other states. A comparison of recommendations by levels of coronavirus positivity and rates of unmet mental health services need is provided in Table 6 below. In all four subgroups of states, emphasis was placed on continued use of telehealth. As stated by a participant from one low positivity/high unmet need state, *“I’m not sure if it’s state to state or state to federal, but it is maintaining the ability of Telehealth services. Probably, this is an issue for CMS (Center for Medicaid Services), that it is a valuable service and should be allowed.”* In states with low unmet need, emphasis was also placed on adopting evidence-based best practices for treatment and documentation of outcomes.

However, each group had their specific set of priorities, such as addressing mental health determinants, consequences and prevention in the context of COVID (high positivity/high unmet need); an emphasis on communication and collaboration with clients and their families and with providers and other stakeholders (low positivity/high unmet need); implementation of universal screening and other evidence-based practices (high positivity/low unmet need); and giving support to clients and their families, providers and agency staff (low positivity/low unmet need).

## 4. Discussion

The COVID-19 pandemic created numerous challenges to the delivery of mental and behavioral health services throughout the United States. Throughout the country, the need for mental health services increased as youth experienced family stress, isolation from peers and home confinement, online learning, fear of illness, and loss of a way of life [5,6,7]. However, this study found that the demand for services was somewhat muted as providers and organizations lost one of the most important sources of referrals for services, educational systems that went online, thus depriving teachers an opportunity to observe student behavior face-to-face. At the same time, the supply of services was impacted by the reduced availability of out of home services like hospitalizations and respite care due to social distancing (i.e., fewer beds) and staff reductions due to infection, fear of infections, or layoffs. The pandemic also impacted the quality of services delivered due to impacts on providers as they struggled to cope with increased levels of family stress and challenges of working at home. As coronavirus positivity rates fueled the need for services, the pre-pandemic levels on unmet service need and persistent staff shortages exacerbated the impacts of the pandemic on supply of services. Increased need, fewer referrals, and reduced supply further combined to increase disparities in services access.

The COVID-19 pandemic also had a significant impact on the development and implementation of child and adolescent mental health services policy. Other studies have documented similar themes related to changes in substance use treatment policies by state single agencies for substance use services (SSAs) [27]. Our study found SMHAs were attempting to implement two different types of policies during this period: policies that responded to the immediate crises in service delivery caused by the pandemic and policies that addressed pre-pandemic issues such as suicide prevention and workforce development that were exacerbated by the pandemic. Apart from guidance provided by the Center for Medicaid Studies [28] and the Substance Abuse and Mental Health Services Administration [29], SMHAs responded to the crisis “on the fly” as described by one participant, relying on questionable data collected from emergency response services and anecdotal reports of increased need for all forms of mental and behavioral health services. This was in contrast to the development and implementation of pre-pandemic policies that utilized research evidence obtained from intermediary organizations, expert consultants, conferences, and professional networks [10,20].

Despite these challenges, the SMHAs of almost all of the states participating in this study reported a rapid pivot to the use of telehealth for services delivery, usually within a matter of weeks. This is consistent with the practice of telepsychiatry in general since the beginning of the pandemic where the implementation of innovations that once took months if not years now occur in a matter of days [30]. Such a pivot was not without its challenges, including limited access to technology and the internet, especially in low income and rural areas, family preference for face-to-face services, lack of privacy, difficulty using with young children and youth in need of substance use treatment, finding a HIPAA-compliant platform, training providers and clients in use, and reimbursement challenges. Preference for face-to-face services and difficulty using telehealth with young children was also reported in a study by Hoffnung and colleagues [31].

SMHAs and other state-level policy makers responded to these challenges by implementing a series of policies designed to facilitate compensation for virtual services, removed barriers to telehealth usage, and license providers to deliver virtual services. As with telepsychiatry in general, rules around Medicare and Medicaid reimbursement, such as Medicare location requirements, were loosened to support and encourage videoconferencing and telephone-based services. Many states implemented COVID-19–specific exceptions no longer requiring psychiatrists and other mental health clinicians’ licensure in the state where a patient is physically located during a video session [30]. These policies were based on policy maker access to the research evidence relating to telehealth effectiveness, data collected by their own agency or other state agencies, and feedback from other policy makers and service providers. Once implemented, these policies resulted in substantially fewer appointment cancellations or no-shows, greater family engagement, reduction in need to the amount of time traveling for services and reduced risk of infection, increased access for people living in remote locations, and increased provider communication and collaboration. Adolescents appeared to prefer telehealth to in-person services delivery [32,33], and states with high rates of coronavirus positivity and high rates of unmet need were most likely to continue use of telehealth post-pandemic. Similar benefits have been reported in studies of use of telehealth to deliver mental health services to adults by primary care providers [34] and specialty mental health care providers [35] during the pandemic.

There appear to be three key elements that influenced the translation of policy into practice during the pandemic. The first element has occurred at the federal level with the granting of Medicaid waivers, the use of Paycheck Protection Program and CARES Act funds to support financially strapped health care systems and providers, and the granting of exemptions to HIPAA regulations. The second element occurred at the state level and was distinguished by pre-pandemic levels of unmet need for services, statewide responses to the pandemic and their impact on positivity rates, and the level of communication and collaboration between SMHAs and public health, child welfare, education, and juvenile justice systems of care. The third element occurred at the local level and was distinguished by provider engagement with SMHAs that identified needs and evaluated solutions to the challenges created by the pandemic, and by provider ingenuity and creativity in telehealth implementation. Each of these elements align with the principles and practice of implementation science [11,12,13,14] and reflect variations in the characteristics of the outer and inner context affecting policy implementation among the different states represented [24,25].

However, the extent to which these activities have impacted disparities in access to mental health services is unclear. Health and mental health disparities associated with COVID-19 have been well documented [36,37,38]. Our previous study found that most (71.5%) policy makers perceived the COVID-19 pandemic as having disproportionately negative mental health impacts on socially disadvantaged youth [15]. Disparities in access to telehealth services related to location (rural) and socioeconomic status (lower income) have also been documented during the pandemic [39,40]. In this study, SMHAs were engaged in several efforts to reduce disparities in access to telehealth, including implementing greater flexibility in regulations and reimbursement for services, providing funding for technology, expanding broadband access, training of providers and consumers, and insisting that telehealth services be evidence-based. Nevertheless, the results also suggest that while these efforts have led to improved access to some populations, especially those in rural areas, it may have also exacerbated disparities in access in other populations such as the urban poor and communities of color. In addition, although our earlier study found no significant concerns with the supply of services [15], some of the participants of this study felt that not all youth were getting the services they needed. Further research is required to assess the overall impacts of the pandemic and shift to telehealth services on mental health equity.

The findings in this study must be interpreted within the specific context where this study took place and how the data were collected. Although we provided numbers of participants who mentioned a particular subtheme or topic, numbers alone do not necessarily reflect its salience or importance to all of the agencies represented in this study [41]. In some instances, a subtheme or topic may have been mentioned by only one study participant; yet its salience or significance must be placed in context of the larger theme in which it was clustered or categorized. Further, although the states were randomized to ensure generalizability of findings, the qualitative information collected in this study was intended to provide a depth of understanding of the phenomenon that was not possible in a quantitative survey and may help to explain some of the discrepancies found in this study and in our earlier study [21]. Second, study participants included representatives of agencies and organizations responsible for development and implementation of mental health services policy. Providers were not interviewed for this study; thus, their perspectives on the extent to which translation of policy into practice was successful is not reflected in this paper. Third, with few exceptions, reports of the increased need for mental and behavioral health services among children and adolescents were largely anecdotal in nature. None of the SMHAs interviewed provided any data demonstrating the effectiveness of virtual services delivery compared to face-to-face delivery. Further, given the rapid assessment design, information on changes in needs and resources over time were retrospective and reflected different points in time during a rapidly evolving pandemic, approximately 6–8 months into the pandemic in the United States.

## 5. Conclusions

Despite these limitations, the findings have several implications for policy implementation during future pandemics as well as non-pandemic conditions. First, they illustrate the need for collaboration and communication across federal, state, and local levels of policy making and implementation and the establishment of formal or informal networks vertically and horizontally to ensure rapid coordination of policies and services during crises. Second, moving forward, a hybrid system of care should be developed for children and adolescents with mental health problems that incorporates both face-to-face and telehealth services. Third, to make such a hybrid feasible, current policies intended to provide flexibility with respect to reimbursement, licensing, privacy and supervision during the emergency should become permanent. Finally, states should be supported in establishing systems of routine data collection to assess levels of need, demand and supply of services as well as service outcomes. Such information is critical to data-driven decision making by SMHAs.

## Figures and Tables

**Table 1:** Demographic characteristics of study participants.

Characteristic	Number	Mean/Percent
Age	29	49.2
Gender		
Male	7	24.1
Female	22	75.9
Race/ethnicity		
Asian	2	6.9
Black	2	6.9
White	24	82.8
Latinx	1	3.4
Education		
Bachelor’s	7	24.1
Master’s	18	62.1
Doctorate	4	13.8
Position		
State Agency Director	2	6.9
Deputy Director	5	17.2
Division Director	8	27.6
Assistant Director	2	6.9
Program Manager	9	31.0
Program Specialist	3	10.3

**Table 2:** Mean rates of coronavirus positivity and unmet need for mental health services for children and adolescents.

Group	Number of States	Mean Positivity Rate	Mean Rate of Unmet Need
1. High positivity/High unmet need	6	9.28 (2.47)	52.37 (4.21)
2. Low positivity/High unmet need	4	4.51 (1.36)	52.20 (6.14)
3. High positivity/Low unmet need	4	8.06 (0.99)	44.67 (3.13)
4. Low positivity/Low unmet need	7	4.11 (1.42)	42.70 (3.83)

**Table 3:** Increase and decrease in demand for mental health service by rates of coronavirus positivity and unmet need for child and adolescent mental health services.

Increase in Demand for Mental Health Services							
	Unmet Need for Child and Adolescent Mental Health Services						
		High (*n* = 10)		Low (*n* = 11)		Total	
**Coronavirus positivity**		*n*	%	*n*	%	*n*	%
	High (*n* = 10)	6	100.0	2	50.0	8	80.0
	Low (*n* = 11)	2	50.0	3	42.9	5	45.4
	Total	8	80.0	5	45.4	12	61.9
**Decrease in Demand for Mental Health Services**							
	**Unmet Need for Child and Adolescent Mental Health Services**						
		**High (*n* = 10)**		**Low (*n* = 11)**		**Total**	
**Coronavirus positivity**		*n*	%	*n*	%	*n*	%
	High (*n* = 10)	4	66.7	3	75.0	7	70.0
	Low (*n* = 11)	1	25.0	4	57.1	5	45.4
	Total	5	50.0	7	63.6	12	57.1

**Table 4:** Decrease in capacity to deliver mental health service by rates of coronavirus positivity and unmet need for child and adolescent mental health services.

	Unmet Need for Child and Adolescent Mental Health Services						
		High (*n* = 10)		Low (*n* = 11)		Total	
		*n*	%	*n*	%	*n*	%
**Coronavirus positivity**	High (*n* = 10)	4	66.7	2	50.0	6	60.0
	Low (*n* = 11)	3	75.0	4	57.1	7	63.6
	Total	7	70.0	6	54.5	13	61.9

**Table 5:** Reported barriers to telehealth by levels of coronavirus positivity and unmet need for child and adolescent mental health services.

	State Group				
	High Positivity High Unmet Need	Low Positivity High Unmet Need	High Positivity Low Unmet Need	Low Positivity Low Unmet Need	Total
	*n*	*n*	*n*	*n*	*n*
Limited internet access	4	3	3	7	17
Limited access to technology	4	3	4	8	19
Family/client reluctance to use	3	2	0	4	9
Privacy	0	2	3	3	8
Hard to use with young children	1	2	0	4	7
Cannot provide certain services	2	2	0	2	6
Virtual fatigue	1	0	2	2	5
Client/provider lack of familiarity	1	0	2	2	5
Provider reluctance to use	1	0	1	1	3
Reduced session duration	0	0	1	2	3
Billing for services	1	0	1	0	2
Getting parental authorization	1	0	1	0	2
State regulations	1	0	0	0	1
Working with schools	1	0	0	0	1
Lack of funding	0	0	0	1	1
Number of barriers	21	14	18	36	89
Number of barriers per state	3.50	3.50	4.50	5.14	4.24

**Table 6:** Recommendations by levels of coronavirus positivity and unmet need for child and adolescent mental health services.

	High Positivity/High Unmet Need	Low Positivity/High Unmet Need	High Positivity/Low Unmet Need	Low Positivity/Low Unmet Need
Telehealth	Continue use of telehealth Use telehealth for crisis services Incorporate telehealth services in schools Provide training in use of telehealth	Continue use of telehealth Conduct telehealth awareness campaigns	Continue use of telehealth Make telehealth available to Medicaid families Reach out to differentially impacted communities Develop evidence-based best practices	Continue use of telehealth Decide what service components can be done virtually Make it easier for families to get telehealth services Develop evidence-based best practices
Other recommendations	Focus on prevention Understand social determinants of health Understand trauma	Regular communication with providers Good staff supervision Give weight and value to youth and family voice and choice More collaboration between state agencies Establish a statewide system of relationships and networks and collaborations	Establish universal screening	Provide peer support Support providers and educators Avoid staff burnout Practice masking and distancing when seeing kids outside Disseminate knowledge of mental health More collaboration between state agencies Adopt other than a formal mental health approach to addressing youth needs during the pandemic Highlight resilience of children and families

## Data Availability

The data presented in this study are available on request from the corresponding author.
